# Pathogenesis of Cognitive Dysfunction in Patients with Obstructive Sleep Apnea: A Hypothesis with Emphasis on the Nucleus Tractus Solitarius

**DOI:** 10.1155/2012/251096

**Published:** 2012-01-16

**Authors:** Mak Adam Daulatzai

**Affiliations:** Sleep Disorders Group, EEE Department, MSE, The University of Melbourne, Parkville, VIC 3010, Australia

## Abstract

OSA is characterized by the quintessential triad of intermittent apnea, hypoxia, and hypoxemia due to pharyngeal collapse. This paper highlights the upstream mechanisms that may trigger cognitive decline in OSA. Three interrelated steps underpin cognitive dysfunction in OSA patients. First, several risk factors upregulate peripheral inflammation; these crucial factors promote neuroinflammation, cerebrovascular endothelial dysfunction, and oxidative stress in OSA. Secondly, the neuroinflammation exerts negative impact globally on the CNS, and thirdly, important foci in the neocortex and brainstem are rendered inflamed and dysfunctional. A strong link is known to exist between neuroinflammation and neurodegeneration. A unique perspective delineated here underscores the importance of dysfunctional brainstem nuclei in etiopathogenesis of cognitive decline in OSA patients. Nucleus tractus solitarius (NTS) is the central integration hub for afferents from upper airway (somatosensory/gustatory), respiratory, gastrointestinal, cardiovascular (baroreceptor and chemoreceptor) and other systems. The NTS has an essential role in sympathetic and parasympathetic systems also; it projects to most key brain regions and modulates numerous physiological functions. Inflamed and dysfunctional NTS and other key brainstem nuclei may play a pivotal role in triggering memory and cognitive dysfunction in OSA. Attenuation of upstream factors and amelioration of the NTS dysfunction remain important challenges.

## 1. Introduction

Obstructive sleep apnea syndrome (OSA) is characterized by the upper airway instability during sleep, reduction or elimination of airflow (hence oxygen desaturation), periodic arousals (hence sleep disruption), and daytime hypersomnolence. About 40% of adults are habitual snorers. The prevalence of OSA has been estimated to be 24% in men and 9% in women [1]. The male : female ratio of the OSA patients has been reported to range from 4 to 1 to 4 to 2 [2]. OSA therefore is a major intrinsic sleep disorder. The alarming degree to which OSA is clinically diagnosed in middle-aged men and women makes it a significant public health problem, and increasing evidence indicates that untreated OSA can lead to several comorbid disorders. OSA is a risk factor for cardiovascular disorders including hypertension, congestive heart failure (CHF), myocardial ischemia, arrhythmias and infarction, and cerebrovascular conditions including stroke [3]. The normal physiologic interactions are disrupted by OSA, and the cardiovascular and cerebrovascular systems are therefore impacted [4, 5]. Overnight polysomnography (PSG) is the gold standard for the evaluation of sleep-related breathing disorders. Apnea-hypopnea index (AHI) is the number of apneic and hypopneic events per hour of sleep. These nocturnal respiratory disturbances result in brief arousals from sleep (i.e., sleep fragmentation) that considerably disturb sleep architecture and may lead to a significant deprivation of rapid eye movement (REM) sleep and stages 3 and 4 of nonrapid eye movement (NREM) sleep. Sleep disturbances and hypoxemia contribute to excessive daytime sleepiness—a common symptom of the syndrome. Approximately 1 in 5 adults possess an AHI of 5–15, that is, mild OSA, and 1 in 15 adults may have moderate OSA, that is, 15–30 AHI [3]. A retrospective study of a cohort of 1,010 patients (844 males, 166 females; similar BMI) found that the AHI in NREM sleep was higher in men than in women (42.9 ± 28.9 versus 32.6 ± 28.7); however, in REM sleep, AHI was similar in men and women (36.0 versus 34.9) [6].

 Nocturnal hypoxia in OSA is a major pathological factor associated with cardiorespiratory diseases [3, 7]. In normal physiologic sleep, distinct sleep stage-related changes occur in cardiovascular regulation. There is a progressive decrease in sympathetic activity, blood pressure (BP), stroke volume, heart rate, cardiac output, and systemic vascular resistance, during deeper NREM sleep stages [4]. However, REM sleep is characterized by increased sympathetic drive; BP and heart rate on average are similar to levels noted during wakefulness [4]. Repetitive apneic episodes disrupt the normal physiologic function and trigger sympathetic activation, vascular endothelial dysfunction, increased oxidative stress, inflammation, increased platelet aggregability, and metabolic dysregulation. During intermittent apneic episodes, hypoxemia and CO_2_ retention activate chemoreflexes and there is vasoconstriction [5]. The above contributory factors impact on the neural and circulatory responses. At apnea termination, there is resumption of breathing, increased cardiac output, and the inhibition of sympathetic vasoconstriction [5].

 Apart from obesity, another problem having enormous impact on society is sleep-disordered breathing—notably OSA. Our knowledge of the upstream factors responsible for the pathogenesis and underlying etiology of memory decline in OSA is still unclear. Animal experiments have shown that intermittent hypoxia for just three days in rats produced memory impairments [8]. Nocturnal chronic intermittent apnea and hypoxemia upregulate humoral, metabolic (including thrombotic), neural, and proinflammatory mechanisms in OSA patients. All of these are, however, known to be associated with the vascular pathophysiology. There is copious evidence to suggest that cerebrovascular pathology/neuroinflammation in patients with OSA may conceivably contribute to the initiation and progression of cognitive dysfunction; furthermore, AD neuropathogenesis may be facilitated by hypoxia. The emphasis, therefore, in the current paper is on linking OSA, neuroinflammation, oxidative stress (generation of reactive oxygen species, ROS), coagulation, metabolic disturbances, and dysfunctional brainstem nuclei such as nucleus tractus solitarius (NTS) and hypoglossal in triggers of cognitive dysfunction. The delineation of the above association may lead to a better understanding of the pathogenetic factors that underpin cognitive decline; this obviously has important therapeutic implications [9, 10].

## 2. Synergistic Pathological Stigmata in OSA

### 2.1. OSA and Metabolic Dysfunction

Models of intermittent hypoxia have significantly improved our understanding of the metabolic impact in OSA. Intermittent hypxia in mice is associated with metabolic dysfunctions, including dyslipidemia, insulin resistance, and pancreatic endocrine dysfunction, similar to those observed in human OSA [11]. OSA is a well-known risk factor for metabolic perturbations and susceptibility to cardiovascular risk and weight gain [3, 12]. Higher leptin (from adipocytes) levels reflect resistance to the normal metabolic effects (appetite suppression and satiety) of this hormone in OSA patients and obese persons [12]. Leptin may predispose to increased cardiovascular risk including platelet aggregation also [13]. Independent of body weight, OSA patients are known to possess higher levels of fasting blood glucose, insulin, and glycosylated hemoglobin [14]. The severity of OSA correlates with the degree of insulin resistance [15]. Although CPAP may reduce leptin levels and decrease visceral fat accumulation [16], glucose tolerance is not improved invariably. This aspect is worth noting since impaired glucose tolerance in OSA patients may be linked to sympathetic activation, leptin resistance, and sleep deprivation [17–19].

### 2.2. OSA and Coagulation

The relatively higher level of the primary fibrin degradation product—PAI-1—and lower level of the primary fibrin degradation product—D-dimer—across the 24h period in OSA patients reflects evidence for a prothrombotic state in OSA [20]. A decreased fibrinolytic capacity and elevated nocturnal levels of catecholamines in OSA may enhance platelet aggregability. OSA may be causally related to increase in clotting activity [21] owing to the documented increases in fibrinogen [22], blood viscosity, and hematocrit [23]. CPAP therapy, however, may reduce platelet aggregability in conjunction with downregulation of catecholamine levels [24] and factor VII clotting activity [25].

### 2.3. OSA and Oxidative Stress

There is persuasive evidence from both animal and human studies for an association between hypoxia and upregulation of oxidative damage [2, 3, 21, 26, 27]. ROS are generated during intermittent hypoxia and reperfusion during repetitive episodes of nocturnal apnea. In OSA, repeated intermittent arterial oxygen desaturation and reoxygenation and ischemia-reperfusion injury to the vascular wall trigger ROS generation [28].

### 2.4. Inflammation

Hypoxia is implicated in the production of inflammation [21, 29] and hence increased levels of inflammatory cytokines, for example, IL-6, TNF-*α*, and of C-reactive protein (CRP) [21, 30]. CRP may play an important role in inhibiting nitric oxide synthase [31], enhancing cell adhesion molecule expression [32], thereby contributing to cerebrovascular disease. Several sources of proinflammatory cytokines and neurotoxicity may occur in OSA patients. These may include (1) obesity [33], (2) infection [34], (3) psychosocial stress [35–37], (4) alcohol abuse [38], and (5) aging itself (see below). Inflammation decreases the efficiency of the capillary system and oxygen supply to the brain, thus reducing metabolic function and oxygen intake in neurons. The above risk factors can exert synergistic and additive actions and increase systemic and brain cytokine production in neuroinflammation.

#### 2.4.1. Aging-Altered Immune Function in Aging/OSA

Aging is characterized by alterations in several functions, including an altered immune function and stress response [39]. There is accumulation of DNA damage due to various factors [40], and indeed there is substantial evidence for elevated production of inflammatory cytokines and oxidative stress in aging [41]. Recurring systemic inflammation may occur due to heterogeneous risk factors—including aging-related diseases, smoking, trauma, infection, psychosocial stress, and indeed sleep disordered breathing. The spatiotemporal interplay of acute and chronic bouts of the above risk factors may be critical in the upregulation/transactivation of inflammatory gene expression at different stages of aging, malnutrition, and pathological states. The peripheral inflammatory insult acts as stimulus that produces in tandem the central phase, namely, the “neuroinflammation,” which is characterized by increases in proinflammatory cytokines in the neocortex and brainstem [42]. The proinflammatory state has an impact on the microglia and switches them to a primed phenotype to synthesize proinflammatory cytokines [43–45]. Consequently, neuroinflammation and the glia-related proinflammatory cascades continue as slow, of low level, progressive, nevertheless of relentless pathology that brings about gradual neuronal degeneration.

 Elderly subjects possess poor physiological reserve and stress tolerance; ongoing cascades of inflammatory response would perpetuate various pathologies including vascular endothelial cell damage, vascular permeabilization, hypotension, and indeed myocardial depression [7]. LPS-induced expression of IL-1*β*, ICAM-1, and IL-6 genes is significantly prolonged and augmented in the aged mice compared with young mice [46]. Several stresses, namely, ischemia due to endotoxin and/or hypoxia may be inducers of gene activation for the expression of the IL-6, ICAM-1, and other cytokines [47, 48]. The synthesis of proinflammatory cytokines exceeds the anti-inflammatory mediators such as IL-10; there is evidence that in the ageing brain anti-inflammatory mediators such as IL-10 are decreased [49]. The inflammatory pathology may be unstoppable or irreversible—unless the peripheral inflammatory insults are stopped and the central neuroinflammation ameliorated [50].

 Studies on the human endothelial cell (EC) cytotoxicity have shown that, following stimulation of ECs with TNF-*α*, the EC toxicity increases greatly [51]. Importantly, age-related oxidative stress itself is sufficient to promote vascular inflammation even in the absence of other well known risk factors such as hypertension or metabolic diseases [52]. Recent experimental data suggest that ROS, innate immunity, the local TNF-*α*-converting enzyme (TACE), and the renin-angiotensin system may underlie NF-*κ*B induction and endothelial activation in aged vasculature; thus, multiple proinflammatory pathways may converge on NF-*κ*B to enhance transcriptional activity of NF-*κ*B during aging [52]. Peripheral inflammation can affect brainstem regions via the circumventricular organs, vagal afferents, and the brain endothelium [53]. Indeed LPS can directly activate the brain endothelium [54], and/or systemic administration of proinflammatory cytokines can cause inflammatory changes in the CNS [53, 55]. A large number of studies have shown neurodegenerative changes in the brains of older adults. Even high-functioning older individuals possess some cognitive complaints (similar to pre-MCI CDR = 0.5) and gray matter atrophy [56–59]. It is posited that most aged persons in fact may harbor circulating peripheral inflammatory cytokines and some neuroinflammation in the neocortex and brainstem nuclei including the NTS, nucleus ambiguous, and DMNV; these nuclei have been documented to undergo aging-related gray matter loss [60]. Consequently, those suffering from long-term snoring/OSA and neuroinflammation could conceivably manifest cognitive deterioration.

#### 2.4.2. OSA/Snoring and Inflammation

Snoring and sleep apnea resulting from incomplete obstruction of the UA may exist in up to 40% of the general population. Nonapneic snorers are known to possess narrower UA. Major independent risk factors for development of habitual snoring are male gender, age between 40 and 64 years, obesity, use of alcohol, sleep medications, and cigarette smoking [61]. The mechanism of snoring is vibration of anatomical structures in the pharyngeal airway, causing high-frequency oscillations of the soft palate, pharyngeal walls, epiglottis, and the tongue. The snoring vibrations have been shown to cause pathologic lesions of the UA mucosa, pharyngeal muscles, and their innervating nerves [62]. The UA mucosa in snorers is edematous, inflamed, and hyperplastic. The levels of proinflammatory cytokines TNF-*α* and IL-6 are elevated in the uvula of nonapneic snorers, although they are much higher in apneic patients [63]. Snoring is associated with cytokine release from blood cells and promotion of inflammation [64]. PCR measurement of mRNA showed that vibration induced a significant expression of proinflammatory cytokines [65]. In a cell model of snoring-induced airway inflammation, mechanical vibration simulating snoring triggered an inflammatory cascade in human bronchial epithelial cells, reflected by the increase in IL-8 release mediated by MAPK pathways [66]. Finally, BMI, alcohol consumption, and cigarette smoking have been repeatedly confirmed to be positively associated with habitual snoring; these are well known to provoke inflammation in both men and women [67]. A recent detailed study found higher CRP, IL-6, and lysozyme in subjects with AHI ≥15 compared with low AHI controls. In multiple linear regressions adjusted for age, waist circumference, and smoking, independent correlation was shown between IL-6 and TNF-*α* and intermittent hypoxia [68].

 Recently, pharyngeal lavage (PHAL) was utilized as a new tool to evaluate pharyngeal mucosal inflammation in OSA patients with and without snoring. PHAL showed lymphocytic inflammation of the pharynx in OSA patients, with neutrophil predominance. Snoring OSA patients had significantly increased numbers of lymphocytes (3.2% compared to nonsnoring OSA and controls group (0.5% and 0.6%, resp.). The cellular infiltration was in accordance with severity; patients with moderate to severe OSA had significantly higher numbers of lymphocytes compared to patients with mild OSA [69].

#### 2.4.3. OSA/Snoring: Cigarette-Smoking-Induced Inflammation

Cigarette smoke produces UA inflammation and edema and is a major contributor to habitual snoring. Habitual snoring was reported in 19.8% of even passive smokers (independent of obesity and sex). A strong link between passive smoking and habitual snoring has been documented in children and adults [70]. Worth noting is that the risk for snoring is four times higher due to smoking than obesity (17% versus 4.3%) [61]. Smoke-induced inflammatory damage affects mucosa including its neural component [71].

#### 2.4.4. Sleep Deprivation and Inflammation

Although sleep deprivation is not a component of stigmata related to OSA, it has been included here since it promotes inflammation. Sleep deprivation may contribute to weight-gain/obesity [72] and promote ROS risk, heart disease, and diabetes [72–74]; these factors potentiate the CNS dysfunction. Even in healthy subjects, sleep deprivation causes a 50% decline in vasodilation reflecting reduced endothelium-dependent NO availability [75]. Proinflammatory cytokines CRP, IL-6, TNF-*α*, and platelet adhesion/coagulation cascade are elevated due to sleep deprivation [76, 77].

#### 2.4.5. OSA and Vascular Endothelial Dysfunction/Inflammation

Vascular endothelial dysfunction reflects a loss of normal homeostatic functions in the macro- and microvasculature. This dysfunction encompasses reduced vasodilation and enhanced vasoconstriction functions, as well as inflammatory/prothrombotic activity. There is extensive evidence for endothelial dysfunction in OSA [78–80].

Hypoxia, hypercapnia, and pressor surges are potent stimuli for the release of vasoactive substances and for impairment of endothelial function during obstructive episodes. Increased levels of endothelin in response to the hypoxemia may promote sustained vascular changes such as vasoconstriction in OSA [81]. Indeed, OSA patients free of any other overt cardiac or vascular disease have been shown to possess impaired endothelial function [82]. Endothelial dysfunction is prominent in smokers [83] and subjects having hypertension [84], hyperlipidemia [85], and diabetes [85, 86]. OSA patients may possess one or all of the above conditions; therefore, OSA alone or in conjunction with comorbidity may result in overt endothelial dysfunction [87].

 Normal endothelium plays an important role in regulating vasomotor tone and maintaining inflammatory and coagulation homeostasis; however, these functions are altered in OSA patients [82, 88]. In OSA, there is increased endothelial cell apoptosis with concomitant impaired endothelial repair capacity [88, 89]. Endothelial dysfunction in conjunction with increased sympathetic activity is implicated in the development of cardiovascular dysfunction of OSA [90, 91]. The reason for endothelial dysfunction in OSA is considered to be due to repetitive hypoxia/reoxygenation during apnea and hypopnea [92]. Despite perfusion during apneas, there is an increase in ROS/inflammation and reduced NO availability in OSA patients [82, 92, 93]. Reduced NO availability in OSA impacts endothelial function and enhances vulnerability for vascular diseases [88]. OSA is characterized by (a) chronic systemic inflammation evidenced, for example, by elevated levels of plasma CRP [30, 94], soluble adhesion molecules [95], and leukocyte superoxide [30, 95], and (b) vascular inflammation noted by upregulation of cyclooxygenase-2 (COX-2) and inducible NOS in endothelial cells [88]. In OSA, aggregation and adhesion of circulating leukocytes to the vascular endothelium may cause blood vessel inflammation [96]. Increased expression of adhesion molecules CD15 and CD11c from monocytes in OSA patients [95] has been implicated in adverse effect on diurnal vascular proinflammatory/antiinflammatory homeostasis [95]. The above has been confirmed by increased production of proinflammatory cytokine IL-4 and a decreased production of an antiinflammatory cytokine IL-10, in patients with moderate to severe OSA [97]. Upregulation of COX-2 in OSA may increase superoxide production resulting in increased oxidative stress, platelet activation, endothelial dysfunction, and vasoconstriction [92, 98–100].

 Finally, an increase in lipid peroxidation and generation of ROS are important features in OSA patients [101, 102] and experimental animals [93, 103]. The reoxygenation phase in OSA is said to be the culprit that promotes ROS production and oxidative stress increase. NO is swiftly scavenged by ROS, producing the toxic metabolite—peroxynitrate. Thus, higher nitrotyrosine expression in endothelial cells depicts enhanced endothelial oxidative stress in the OSA patients [88]. The endothelial oxidative damage and ROS production in conjunction with a decrease in NO perpetuate a cyclical pattern of endothelial injury and inflammation [104]. The above-mentioned scenario may upregulate cell death receptors and mitochondria-dependent apoptotic pathways, culminating in endothelial apoptosis [105].

### 2.5. Pathological Risk Factors and Cognitive Impairment

Even a couple of days of intermittent hypoxia produces detectable spatial memory impairments in rats [14]. There is a large literature showing that each of the cardiovascular risk factors, including abdominal obesity, hypertension, dyslipidemia, and hyperglycemia, is individually associated with cognitive impairment in older adults. In a recent study, up to 20.6% older persons suffered from cognitive impairment, but no dementia (CIND), and hypertension was the significant and independent factor associated with CIND [106]. Cumulative epidemiological evidence emphasizes that vascular and vascular-related factors may be crucial in the development of age-related cognitive decline. It is important to underscore the important pathophysiological link between inflammation (e.g., in metabolic syndrome) and cognitive impairment (of vascular/degenerative origin) [107, 108].

 There is enhanced procoagulant and thrombotic activity in OSA patients [21]. Several studies correlate elevated prothrombotic levels with increased risk for cognitive dysfunction. It has been suggested that the association between amyloid *β* and fibrinogen causes aberrant fibrin hemostasis that could lead to compromised blood flow and increased inflammation, thereby contributing to cognitive decline [109].

 In a recent Baltimore longitudinal study, obesity indices were associated with poorer performance in a variety of cognitive domains, including global screening measures, memory, and verbal fluency tasks [110]. Let alone obese elderly, even obese adolescents may possess disinhibited eating behaviour associated with reductions in orbitofrontal cortex (OFC) volume [111]. Obese subjects have decreased dopaminergic activity and reduction in glucose utilisation in dorsolateral prefrontal (PFC) and orbitofrontal (OFC) areas (areas subserving inhibitory control and salience attribution) [112]. Recent data have also demonstrated reductions in frontal lobe function tests and correlated reductions in OFC volumes in the obese individuals [113].

 As emphasized throughout this paper that systemic inflammatory signals communicate with the brain (causing neuroinflammation) and lead to oxidative stress and other changes in its metabolism. Notably, the primed microglia undertakes enhanced synthesis of proinflammatory mediators. There is copious evidence that neuroinflammation contributes to the exacerbation of acute symptoms of chronic progressive neurodegeneration and cognitive impairment [114]. Various circulating inflammatory mediators may directly or indirectly gain access and influence the activity of NTS neurons. Adjacent to NTS is the area postrema (AP); it lacks a blood brain barrier (BBB), and its prominent axons project to the NTS. The NTS has direct and indirect connections to a wide range of neural structures including the PFC and hippocampus, thus possessing the capacity to affect their physiological processes. The gustatory afferents project to the NTS. The prefrontal neural network including the OFC and medial PFC is one of the pivotal regions for bidirectional functional association between the brain and autonomic and immune activities [115, 116]. A dysfunctional and inflamed NTS (see further discussion of this point in Section 3) would have an adverse impact and perturb the physiological functions of the PFC as well as other key brain regions. Ascending from the NTS, the vagus reaches a large number of cortical and subcortical regions, including the PFC. Chronic oxidative stress plus systemic/neuroinflammation may cause dysfunctional synaptic transmission and attenuate multitude of efferent signaling pathways. The above points have been further delineated in the following sections.

## 3. Disparate Pathophysiologic Mechanisms

Aging is a biological process characterized by time-dependent, progressive, physiological decline including attenuated CNS functions of sensory, motor, and cognitive modalities. Aging is accompanied by increasing incidence of age-related diseases such as OSA and Alzheimer's disease (AD). Inflammation is considered pivotal in age-related physiological alterations and pathogenesis of many age-related diseases, owing to a wide variety of inflammatory mediators mentioned above. Aging has been suggested to be a state of chronic, low-grade molecular inflammation which may trigger the pathogenesis of several diseases [117, 118]. In this regards, available data have established two facts: (1) aging-associated dysregulation of the immune system and (2) aging-associated alteration of redox status. Both processes intertwine and exacerbate systemic inflammatory status. Several studies have highlighted an increased inflammation in old age [119, 120]. Glial cells from old mice also secrete more proinflammatory IL-6 and less of anti-inflammatory IL-10, compared to young adults [121]. An insidious close relationship exists between systemic infection/inflammation and cognitive dysfunction in the aged [119, 122]. Stimulation of the peripheral innate immune system (e.g., with lipopolysaccharide, LPS) causes increased neuroinflammatory response in the brain of aged mice [123] and humans [124]. Aged animals undergo neuroinflammatory alterations whether LPS is injected directly into the brain or into the systemic circulation. Old animals infected with *Escherichia coli* possess increased hippocampal interleukin IL-1*β* and several other inflammatory cytokines and undergo deficits in hippocampus-dependent memory, in comparison with similarly infected younger animals [125]. This is because of inherent propensity in aging—in that systemic circulating inflammatory cytokines (CIC) impair synaptic function/plasticity [43, 126] and may decrease gray matter volume in the hippocampus [127, 128] and brainstem nuclei [60]. There is strong clinical evidence that AD is associated with an inflammatory response, particularly due to higher peripheral concentrations of IL-6, TNF-*α*, IL-1*β*, IL-12, and IL-18 [129]. Consequently, an increase in neuroinflammatory response is fundamental in being correlated with susceptibility to cognitive impairment.

### 3.1. Afferent Dysfunction in OSA

The UA anesthesia increases pharyngeal airflow resistance and can induce or increase apneas and hypopneas in normal subjects and snorers [130–132]. Other data also suggest that impairment of sensory receptor function could conceivably produce sleep apnea [132, 133]. Thus, interruption of an afferent feedback mechanism, that is, of sensory stimuli arising in the UA mucosa, leads to apnea. This provides support for the concept that it is the status of afferent stimuli (arising in peripheral receptors) that plays a cardinal role in the patency of UA or its occlusion. In snoring subjects with or without OSA, vigorous snoring-related vibration and repeated forceful suction collapse of the pharynx could be traumatic to the UA mucosa and thus produce inflammation, edema, disturb sensory function, inducing neural damage [62, 134]—analogous to peripheral nerve injury resulting from low-frequency vibration [135]. Neural injury and dysfunction in OSA patients are widespread in several sites causing anatomicophysiological perturbations. This is consistent with data that palatopharyngeal muscle biopsies of OSA patients (undergoing uvulopalatopharyngoplasty) show mucosal edema and neurogenic damage [62, 136]. Indeed, UA mucosal edema has been demonstrated by magnetic resonance imaging [137] in OSA patients. There is also demyelination of motoneurons in palatal tissue in OSA [138]; consistent with this, EMG data on palatopharyngeus muscle in OSA subjects demonstrated long polyphasic potentials and reduced amplitude [139]. Not surprisingly, therefore, proapoptotic proteins including caspases are upregulated following intermittent hypoxia-related mucosal injury [50, 103, 140]. The circulating systemic cytokines, as delineated above, may lead to microglial activation and inflammation-mediated neurotoxicity.

### 3.2. Dysfunctional Circadian Rhythm in OSA

Chronic intermittent hypoxia (CIH), repeated arousals, and irregular sleep-wake rhythm in OSA patients are related to abnormal circadian rhythm reflected in daytime somnolence and overall dyshomeostasis [141]. Studies have emphasized that OSA per se contributes to altered circadian rhythm in autonomic activity and BP thus promoting the cardiovascular diseases [142].

### 3.3. OSA and Cerebrovascular Factors

Sleep is a state in which consolidation of newly acquired information into memory takes place. This process is facilitated by neuromodulatory activity patterns and electric field potential oscillations; NREM and REM sleep support system consolidation and synaptic consolidation, respectively. Reactivation and redistribution of hippocampus-dependent memories to neocortex occur during slow wave sleep (SWS) via slow oscillations, spindles, and ripples [143, 144]. OSA, hypertension, and increased body weight correlate with decreased brain volumes, including the prefrontal cortex and cognitive dysfunction [60, 145, 146]. Thus, perturbations in CNS homeostasis due to disparate risk factors including intermittent apnea impact on sleep-related hippocampal and posterior cortical regions' memory processes [144–146].

 Normally, brain perfusion is a function of tightly coupled metabolic demand and oxygen availability. A major pathological factor in OSA is nocturnal hypoxia; the resultant hypoxemia is deemed to impose stress on the brain, in particular. The brain is particularly vulnerable to the hypoxic stress, and chronic nocturnal intermittent hypoxia may directly damage the brain tissue. The pathological loss of neocortical/CNS gray matter due to hypoxia as mentioned above may correlate with impaired cognitive function. Normally, the hypoxic repercussion is mitigated during wakefulness; a decrease in O_2_ supply causes a decrease in cerebral vascular tone and a consequent increase in cerebral blood flow (CBF), being linearly related to the fall in arterial O_2_ saturation (Sa_O2_) [147]. During NREM stage 3/4 sleep, the control of the cerebral vascular system is rather tenuous, in that there is a decrease in both cerebral blood flow and cerebral metabolism [148]. It has been observed that the CBF response to hypoxia is absent during stage III/IV NREM sleep [149, 150]. In response to isocapnic hypoxia, cortical blood flow increases during wakefulness; however, the same degree of isocapnic hypoxia may decrease the cortical blood flow during sleep [149, 150]. Importantly, light sleep (stage II) is characterized by CBF and cerebral oxygen metabolic rate (CMR) reduction by 3–10% (below the level associated with wakefulness), whereas CBF and CMR during deep sleep (stage III-IV) are dramatically reduced by 25–44% [151]. This may explain the possible interrelationship between a reduction in cerebral vascular response to hypoxia. Another potential factor that may be integral to hypoxia-related vasoconstriction is endothelin-1 (ET-1) which is known to exert a potent constrictor action on the cerebral circulation [152]. Plasma ET-1 level measured by radioimmunoassay was significantly increased in the rats having intermittent hypoxia/hypercapnia (IH) [152]. The arteries show increased constrictor sensitivity to endothelin-1 in the hypoxic animals. Finally, in terms of circadian variation, ET-1 levels are highest during the night and in the early hours of the morning [153]. NO promotes cerebral vasodilatation and couples blood flow and brain activity. NO is produced by active neurons and may couple brain activity and blood flow in sleeping lambs [154]. In humans, circulating blood NO levels are reported to be lowest during the night and in the early hours of the morning [153]. Therefore, a reduced endothelial and/or neuronal NO production would be an important factor in reducing the vasodilation of cerebral vasculature, reducing the cortical CBF response and attenuating gray matter volume in OSA patients [155, 156].

### 3.4. Central Inflammation Affects CNS Homeostasis and Promotes Cognitive Decline

Several studies confirm that preexisting inflammation increases vulnerability to a subsequent peripheral immune challenge, thus exacerbating the deleterious effects [125, 129, 157]. Furthermore, it is well documented that peripheral inflammatory cytokines stimulate central inflammatory cytokine mRNA and protein synthesis [157–159]. Aged rats, that exhibit signs of neuroinflammation, are inherently more responsive to the subsequent exposure/effect of LPS or infection. Increase in peripheral cytokines increases synthesis of IL-1*β* in the CNS [158]. Higher levels of proinflammatory cytokine mRNAs for IL-1*β*, IL-6, and TNF-*α* in CNS are induced by the individual/additive effect of the stimulated peripheral cytokines. Consequently, the proinflammatory and anti-inflammatory balance is perturbed, thus causing the activation of different downstream pathophysiological cascades. Following activation, platelets adhere to leukocytes and endothelial cells via p-selectin, platelet endothelial cell adhesion molecule-1 (PECAM), and intercellular adhesion molecule-1 and -2 (ICAM-1 and -2) and secrete phospholipase A2 and cyclooxygenase-2 (COX-2) as well as other proinflammatory chemokines and interleukins [160]. Further, they are a rich source of intraplatelet A*β*-40 [161]. Although platelets promote coagulation, wound healing, angiogenesis, and other functions, they are also essential for the innate immune response to combat infection (viruses, bacteria, microorganisms). They help maintain and modulate inflammation and are a major source of proinflammatory molecules such as P-selectin, tissue factor, CD40L, and metalloproteinases; they are major players indeed in promoting pathologies in several diseases, including AD [161].

 Hypoxia stimulates the expression of inflammatory cytokines (IL-1*β*, TNF-*α*), chemokines (IL-8, MCP-1/CCL2), and adhesion molecules (ICAM-1) in the brain, in cultured astrocytes and in brain endothelial cells [162–166]. Chronic intermittent hypoxia activates several factors including hypoxia-inducible factor-1 (HIF-1), c-fos, activator protein-1, and NF kappaB. Hypoxia-induced HIF-1*α* expression occurs both in tissues and cultured cells [163, 167, 168]. HIF-1*α* is an essential molecule that regulates oxygen homeostasis and mediates hypoxia-induced expression of IL-1*β* in astrocytes [166]. Astroglial cells are the most abundant cells in the brain and play an important role in the initiation and progression of hypoxia-induced neuroinflammation. HIF-1*α* initiates upregulation of inflammatory cytokines; upregulation of inflammatory genes by hypoxia is mediated by different transcription factors including HIF-1, NF*κ*B, and AP-1 [162, 163, 169]. Recent work further demonstrated the role of HIF-1*α* in hypoxia-induced upregulation of inflammatory chemokines, human monocyte chemoattractant protein-1 (MCP-1/CCL2), and mouse MCP-5 (CCL12), in human and mouse astrocytes, respectively [164, 165, 170–172]. Activation of the HIF-1*α* pathway by risk factors such as age, cerebral atherosclerosis, and neuroinflammation may contribute to A*β* deposition and cognitive dysfunction.The above data, therefore, provide an important link for understanding the involvement of OSA and inflammation [173] as upstream mechanisms that may promote the downstream cascades (namely, of A*β* deposition and tau phosphorylation) of neuropathogenesis causing cognitive decline.

### 3.5. Nucleus Tractus Solitarius (NTS): Not Just an Innocent Bystander

The NTS is a compact network of neurons; its copious afferent and efferent pathways affect central homeostatic control [174]. This nucleus contains an enormous range of neuroactive substances; indeed, most of those identified within the CNS are also found in the NTS, as neurotransmitters and neuromodulators [175]. NTS located in the dorsal brainstem is the primary site for termination and integration of sensory afferents, such as baroreceptor, chemoreceptor, nocioceptors, and afferents from several key body systems, including gastrointestinal, respiratory, and cardiovascular, and indeed from UA and tongue. Thus, the NTS is the first CNS region for synaptic contact of the above afferents. The signal processing at these synapses determines the output of the sensory information from the heart, lungs, gut, airways, and the tongue to all downstream NTS synapses in the reflex pathways. The second-order NTS neurons spatially and temporally integrate the sensory information including the vagal afferent inputs, orchestrate an efferent output, and transmit it to various interconnected foci including the hypoglossal nucleus and the parasympathetic preganglionic neurons of the DMNV [176]. There is evidence that inflammatory mediators can influence the brainstem neuronal function directly and the NTS itself is a primary CNS detector of cytokines [177]. Indeed, NTS neuronal function can be affected directly through local synthesis of inflammatory mediators [53]. Thus, binding of cytokines, for example, IL-1*β* to its receptors on the neuronal membrane, initiates signaling cascades upregulating transcription of genes such as COX-2, TNF-*α*, and IL-6; these then recruit leukocytes and macrophages that release additional inflammatory cytokines [53, 178, 179]. There is bound to be an overall general impact of neuroinflammation on several brain regions including the hippocampus [180, 181]; such an impact would not only perturb their functions but would also have an adverse impact on the NTS owing to their reciprocal projection. The efferent parasympathetic pathways constitute the “cholinergic anti-inflammatory pathway” [182, 183]. Ascending from the NTS, the vagus reaches the thalamus, the paraventricular nucleus, the central nucleus of the amygdala, the hippocampus, the insula, the anterior cingulate cortex (ACC), and the medial prefrontal cortex (MPFC) [184]. The NTS provides input to the parabrachial nucleus, the DMNV, and the nucleus ambiguous (NA); these nuclei provide extensive efferent signals [140]. Chronic neuroinflammation may cause dysfunctional synaptic transmission and thus impact many key brain regions adversely, attenuating multitude of efferent signaling pathways of the NTS [37, 185–188]. A dysfunctional NTS would be deleterious to numerous key CNS foci and body systems that project their afferents to this strategically essential nucleus (many with reciprocal connections) [174, 180, 184–188]. Taken together, the abovementioned studies suggest that the dysfunctional NTS has the propensity to promote cognitive disturbances in OSA. Conceivably, a proportion of such elderly may progress to mild cognitive impairment and AD.

 The GMV loss in specific brainstem nuclei in asymptomatic elderly reflects an ongoing silent pathophysiological change [60]. The elderly, suffer from on one hand, subclinical ongoing decreases in olfactory, gustatory, and somatosensory modalities of senescence [189–192] and, on the other, dysfunctional NTS activity due to neuroinflammation mentioned above. This then may lead to further decreases in sensory modalities projecting to the thalamocortical system and the NTS. Since the NTS projects to the hypoglossal nucleus, a decrease in the NTS function could conceivably affect in the direction of low NTS function → low hypoglossal function → low genioglossus activity → decrease in pharyngeal patency, resulting in → intermittent hypoxia/hypoxemia. The recurring hypoxic episodes of OSA may further potentiate pathology of the parietal, temporal, and frontal lobes, and the basal forebrain in the neocortex, and indeed in the key brainstem nuclei. Conceivably then, the neuroinflammation and OSA-related neuropathological alterations may promote cognitive dysfunction. Neuroinflammation, oxidative stress gene activation, and ROS production cause protein, lipid, and nucleic acid oxidation and negatively impact the neuronal homeostasis in the NTS. This conclusion is supported by experimental studies employing ROS/neuronal degeneration approach [193]. Importantly, neuroinflammation, intermittent/episodic apnea/hypopnea, and ROS/oxidative stress may synergize to augment pathology in CNS including apoptosis in the neocortical regions, brainstem, and indeed the NTS [194–198]. Compelling evidence therefore supports OSA/neuroinflammation/oxidative stress explanation in the causation of cognitive pathology—whose epicenter is the multifunctional highly interconnected NTS hub.

 Recent studies have implicated the microvasculature inflammation in brainstem, specifically in the NTS, in the pathogenesis of hypertension [199–205]. It has been shown that vessels within brainstem regions of hypertensive animals (SHR) (an animal model of human essential hypertension) are inflamed and release ROS and cytokines; these pathological messengers then alter neuronal activity in the NTS [201–205]. In the NTS of SHR, the gp39 precursor was upregulated [201]; the gp39 precursor is homologous to chitinase 3-like protein 1, also known as human cartilage-gp39 or YKL40. High levels of this molecule are present in many different inflammatory conditions including rheumatoid arthritis, glioblastoma, inflammatory bowel disease, atherosclerosis, asthma, and indeed AD [206]. Furthermore, gp39 precursor also promotes chemotaxis [207]. Thus, upregulation of gp39 precursor in the NTS reflects an inflammatory state that may attenuate neuronal activity in this brainstem nucleus [201–205]. Furthermore, the brainstem vessel inflammation could conceivably elevate the resistance to blood flow causing inadequate perfusion and exerting deleterious effects on neuronal excitability/viability in the NTS. An inflamed and dysfunctional NTS consequently may cause widespread disruption of many key biological functions in both brainstem and neocortex causing dyshomeostasis.

## 4. Focus on Correlates of Cognitive Dysfunction and the Unifying Hypothesis

### 4.1. Chronic Intermittent Hypoxia and Cognitive Dysfunction

Hypoxia due to OSA has been shown to cause neuropathological changes and memory impairments. Cognitive dysfunction may result due to decreased oxidative metabolism in the brain and impairment of neurotransmission. OSA is associated with unique cerebral alterations that may explain the behavioral and neurocognitive alterations observed. Several data utilizing several different techniques such as transcranial Doppler, event-related potentials, MR spectroscopy, and structural and functional MRI have clearly demonstrated changes in blood flow, metabolism, morphology, and activation in neurocognition-related brain regions in aging and OSA patients [150, 208–210]. Decreased cerebral activation during the working memory task in OSA patients reflects that these individuals possess impaired cerebral responses during executive function [211].

 Compared to healthy subjects, the gray matter concentrations of OSA patients were significantly decreased in the left gyrus rectus, bilateral superior frontal gyri, left precentral gyrus, bilateral frontomarginal gyri, bilateral anterior cingulate gyri, right insular gyrus, bilateral caudate nuclei, bilateral thalami, bilateral amygdale and hippocampus, bilateral inferior temporal gyri, and the cerebellum [156]. Another study exhibited markedly declined signals in the ventral thalamus, hippocampus, and insula in OSA patients, compared to controls [212]. Neuroimaging data have provided evidence of hippocampal atrophy in OSA patients with a linear relationship between hippocampal volume and memory performance [213]. Freshly dissociated hippocampal CA1 neurons, exposed (Cyc) neurons exposed to hypoxia, showed decreased excitability; they showed action potentials (AP) with smaller amplitude and a longer duration and a more depolarized resting membrane potential, compared to controls [214]. Since the hippocampus is particularly susceptible to hypoxia, its bioenergetics is negatively impacted. Indeed, proton MR spectra obtained from the left hippocampus of OSA patients showed lower levels of hippocampal creatine-containing compounds; furthermore, they correlated with worse OSA severity and neurocognitive performance [215]. This further suggests that OSA has the propensity to impact regions that subserve cognitive processes.

Depression is not uncommon in OSA patients. Neural injury differed between OSA patients with and without depressive symptoms. Depressive symptoms accompanying OSA exacerbated injury. When MRI maps were compared between OSA and control groups, injury appeared in symptomatic relative to asymptomatic OSA subjects in the mid- and anterior cingulate, anterior insular, medial prefrontal, parietal, and left ventrolateral temporal cortices, left caudate nucleus, and internal capsule. However, symptomatic OSA patients with depression showed damage in the bilateral hippocampus and caudate nuclei, anterior corpus callosum, right anterior thalamus, and medial pons [216]. Additionally, when objectively measured disturbed sleep was consistently related to poorer cognition, whereas total sleep time was not; thus, it is the disturbance of sleep rather than quantity of sleep that affects cognition [217–219].

 Various data have shown gray matter loss in cognitively relevant brain regions in hypoxia [60, 146, 220]. The above observations are in keeping with cortical neuronal cell damage due to intermittent hypoxia associated with neurocognitive dysfunction [103, 220, 221]. Another mechanism analogous to intermittent hypoxia is the ischemia/reperfusion-related reoxygenation [222], where enhanced ROS generation causes damage [223, 224]. Of note are data on 100 healthy male and female subjects of different age groups; magnetic resonance angiograms (MRA) displayed a lower number of MRA-discernible microvessels in aged individuals [210]. In addition, there was a significant increase in vessel tortuosity with age, limited to the middle cerebral distribution [210]. In healthy aging, OSA is associated with reduction of blood flow from the middle cerebral artery to the cortex that may negatively impact cortical neurons [225, 226]. In several elegant studies, Gozal, Kheirandish, and their colleagues have documented the interplay between OSA, NO, inflammation, and oxidative stress as pathological culprits that cause memory disturbance and neurodegeneration [103, 227, 228].

### 4.2. Episodic Hypoxia, Repercussion, and the NTS

There is significant evidence that OSA is independently associated with metabolic dysfunction, including dyslipidemia, insulin resistance, and overweight/obesity [229]. The latter as well as other factors in OSA may contribute to sleep debt, repetitive hypoxemia, increased sympathetic tone, and indeed hypertension. Recent studies have provided new insights in that OSA affects lipid and glucose metabolism by increasing adipose tissue lipolysis with subsequent free fatty acid flux to the liver, upregulating lipid synthesis in the liver and inhibiting lipoprotein clearance. OSA enhances inflammation and sympathetic activation that may affect glucose metabolism and counterregulatory hormones. Indeed models of OSA have also improved our understanding of the metabolic impact of intermittent hypoxia [230].

 A common neuronal protective adaptive reaction to hypoxic stress (hypoxia and inflammation) is the lowering of the cellular metabolic rate and energy decrease, preventing hypoxic excitotoxicity, and depression of synaptic activity. This could conceivably occur in the stressed NTS neurons exposed to hypoxia and inflammation in OSA patients. ROS enhance cellular inflammatory responses and reduce the expression of genes required to maintain synaptic structure and function. Documented evidence shows that pathological neurons synthesize proinflammatory cytokines and activate microglia [231, 232]. TNF-*α* and IL1-*β* are known to induce each other and their own production; thus, these cytokines may exacerbate the NTS pathology causing its neuronal dysfunction further. Thus, aberrations in NTS neural signaling, in the presence of hypoxia, ROS, and neuroinflammation, may promote neuronal degeneration in brainstem and cortex and lead to cognitive decline.

 In addition to hypoxemia reoxygenation, OSA is characterized by other stressors, including intrathoracic pressure swings, and arousals from sleep, peripheral vasoconstriction, and rises in blood pressure (BP), and indeed inflammation. There is copious evidence from animal and human studies that sympathetic nervous system activation caused by hypoxia and arousals links OSA and BP. An important marker of cardiovascular risk is the vascular endothelium which may be dysfunctional in OSA. The dilatory response of small vessels to vasoactive substances such as acetylcholine may be blunted in sleep apnea [233, 234]. Levels of endothelin, a potent vasoconstrictor, may also be elevated in OSA patients [235]. In addition, bioavailability of NO is reduced in OSA patients owing to decreased eNOS activity and increased nitrotyrosine production (byproduct of nitric oxide degradation) in endothelial cells in this disorder [88]. Heightened inflammation, reflected by elevated C-reactive protein (CRP), leukocyte adhesion factors in OSA [236, 237], neutrophil-derived oxidative stress [238], and abnormalities in coagulation markers in patients with OSA may modulate vascular risk and upregulate predisposition to endothelial injury.

 Episodic deoxygenation/hypoxemia stimulates the peripheral arterial chemoreceptors. Thus, the carotid body afferents, relaying in the NTS, elicit reflex increases in sympathetic efferent function [239, 240]. Each CNS arousal from sleep is accompanied by enhanced sympathetic neural outflow [241] that may exacerbate autonomic dysregulation in OSA. Hence, chemoreflex activation and/or arousal in OSA increases sympathetic drive to the peripheral vasculature and enhances BP [242, 243]. Normally, stimulation of parenchymal vagal receptors in lung (i.e., lung inflation) tempers sympathetic outflow; however, during apneas (i.e., a lack of lung inflation), sympathetic neural activity potentiates sympathetic responses to hypoxia/hypoxemia [244, 245]. The combination of advanced age with increased oxidative stress, hypertension, inflammation, and other risk factors provides a rich background—for altered regulation of blood flow, deposition of amyloid *β* protein and neurofibrillary tangles, altered cholinergic transmission, and autonomic dysfunction. These pathways interact in a complex pattern. For example, changes in BP negatively affect brain perfusion and metabolism, and clearance of amyloid *β* protein from the brain is dependent on vascular reactivity, which in turn is affected by endothelial/vascular injury and remodelling. Presence of comorbidities and multifaceted pathophysiological interactions may result in cortical/subcortical dysfunction, neuronal atrophy/death, and cognitive decline [173].

 In OSA, the dysfunctional NTS/vagal mechanisms are correlated with reduced lung inflation, baroreflex and chemoreflex dysfunction, and dysregulation of cardiorespiratory homeostatic mechanisms. Both cardiac phasic and pulmonary tonic activity of the vagus participate in the function of cardiovascular and respiratory systems and play a pivotal role in coordinating their normal activity. Vagal nerve blockade (by atropine) results in an increased alveolar dead space and reduced *P*_aO2_ and *S*_pO2_. Attenuated vagal activity due to the NTS dysfunction impacts physiological adjustment to improve pulmonary gas exchange efficiency—enhancing hypoxia, hypercapnia, and tracheobronchoconstriction [246]. The above leads to the vicious cycle of dysfunctional parasympathetic phenomena that impact cognitive outcomes; the following current hypothesis is therefore consistent with the above implications.

### 4.3. The Hypothesis

**Figure 1:**
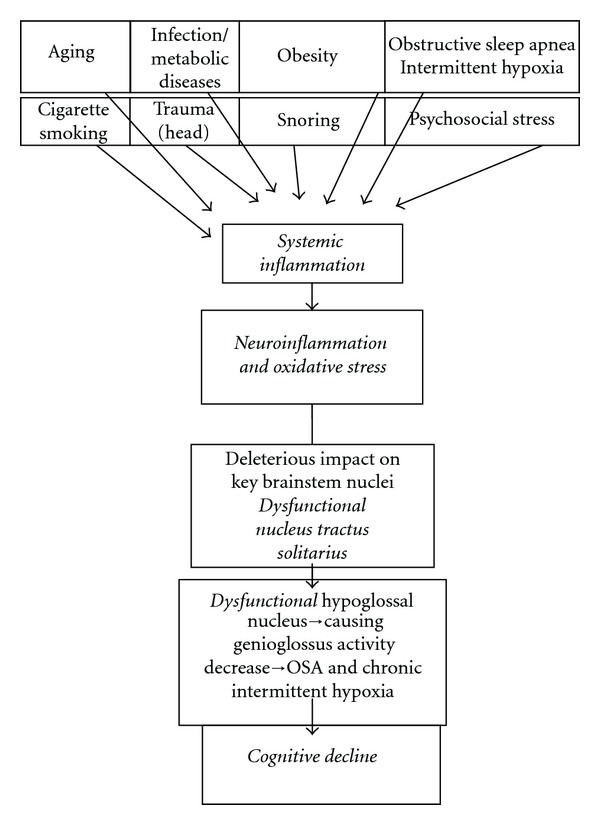
Schematic representation of the pathogenesis of cognitive decline in patients with sleep disordered breathing. Several risk factors upregulate proinflammatory cytokines and induce systemic inflammation. The latter then promotes neuroinflammation which augments further inflammation; this has a negative impact on neuronal, glial, and vascular endothelial cell functions. Inflammation, intermittent hypoxia, and oxidative stress provide a rich milieu causing the NTS inflammation and dysfunction. Being a central hub for processing disparate afferents and owing to its widespread projections in the CNS, the dysfunctional NTS would negatively impact several key brain foci and their physiological functions. Dysfunctional NTS and hypoglossal nuclei would cause genioglossus dysfunction resulting in pharyngeal obstruction/collapse. This leads to snoring, OSA, intermittent hypoxia, and hypoxemia. Thus, neuroinflammation and sleep apnea are major contributing factors that may cause neuronal degeneration and provoke cognitive dysfunction.

Based on the above-mentioned evidence that underscores the role of inflammation and OSA in cognitive disturbances, one can hypothesize highlighting that—aging plus several risk factors generate proinflammatory cytokines/oxidative stress/ROS → Neuroinflammation—deleterious impact on neurons/glia of neocortex and brainstem → brainstem nuclei including the NTS-inflamed and dysfunctional → attenuation of hypoglossal nucleus activity → genioglossus dysfunction promoting snoring/OSA/dysfunctional breathing → chronic intermittent hypoxia and hypoxemia → further neuroinflammation and neuronal degeneration → global deleterious impact on a host of key physiological functions in brain (e.g., in hippocampus) → memory and cognitive dysfunction (Figure 1). The implications of the current perspective are considerable; attenuation of the predisposing upstream risk factors delineated here warrants future research.

## 5. Conclusions

Nocturnal intermittent hypoxia and hypercapnia are cardinal features of OSA. Altered cerebral circulation, brain hypoperfusion/ischemia, and the pathological loss of gray matter are associated with OSA. Aging is accompanied by a low-grade, chronic, and clinically indolent upregulation of proinflammatory state. A simple and potentially pragmatic hypothesis is posited on the cognitive decline in OSA patients. The immune system and inflammation have been implicated in a wide variety of neurodegenerative conditions. Relatively common sources of systemic inflammation may be significant risk factors that may potentiate neuroinflammation in the CNS. The latter causes microvascular changes, switching of microglial phenotype and activity, and physiological dysfunctions in key brainstem nuclei, notably, the NTS and the hypoglossal. Their inflammation and dysfunctional activity result in genioglossus dysfunction, leading to UA obstruction and intermittent hypoxia/hypoxemia. The latter has widespread impact on several physiological activities, including the CNS structure and function—thus further enhancing inflammation and causing grey matter volume decrease. Neuroinflammation plus hypoxia may therefore underpin the CNS physiopathology leading to cognitive dysfunction. The NTS is the central integration hub for afferents from the UA somatosensory/gustatory, gastrointestinal, respiratory, cardiovascular (baroreceptor and chemoreceptor), and several other afferents from the brain (e.g., from amygdale and hypothalamus). It also has important role in sympathetic and parasympathetic systems. The current hypothesis, therefore, implicates inflamed and dysfunctional NTS as the central player in the neuropathogenesis of cognitive decline. The current hypothesis is the first to sequentially connect aging plus several risk factors → generate proinflammatory cytokines/ROS/oxidative stress → dysfunctional NTS and hypoglossal → snoring/OSA/dysfunctional breathing → hypoxia and hypoxemia → further neuroinflammation and neuronal degeneration → global deleterious impact on a host of key physiological functions in CNS → memory and cognitive dysfunction. In some OSA patients, however, this may lead to age-associated dementing disease such as AD. The implications of the current perspective are considerable and warrant future research.
